# Evaluation of the Diagnostic Potential of IP-10 and IL-2 as Biomarkers for the Diagnosis of Active and Latent Tuberculosis in a BCG-Vaccinated Population

**DOI:** 10.1371/journal.pone.0051338

**Published:** 2012-12-14

**Authors:** Sen Wang, Ni Diao, Chanyi Lu, Jing Wu, Yan Gao, Jiazhen Chen, Zumo Zhou, Heqing Huang, Lingyun Shao, Jialin Jin, Xinhua Weng, Ying Zhang, Wenhong Zhang

**Affiliations:** 1 Department of Infectious Diseases, Huashan Hospital, Fudan University, Shanghai, China; 2 People’s Hospital of Zhuji, Zhejiang Province, Zhuji, China; 3 Department of Molecular Microbiology and Immunology, Bloomberg School of Public Health, Johns Hopkins University, Baltimore, Maryland, United States of America; 4 MOH and MOE Key Laboratory of Medical Molecular Virology, Shanghai Medical College, Fudan University, Shanghai, China; 5 Institutes of Biomedical Sciences, Fudan University, Shanghai, China; National Institute for Infectious Diseases (L. Spallanzani), Italy

## Abstract

**Background:**

The *Mycobacterium tuberculosis* (*Mtb*)-specific T-cell interferon gamma release assays (IGRAs) are useful in detecting *Mtb* infection but perform poorly at distinguishing active tuberculosis disease (ATB) and latent tuberculosis infection (LTBI). This study is aimed at evaluating additional cytokines as biomarkers besides interferon-gamma (IFN-γ) to improve the identification of ATB and LTBI.

**Methodology/Principal Findings:**

Sixty-six patients with ATB, 73 household contacts (HHC) of ATB patients and 76 healthy controls (HC) were recruited to undergo QuantiFERON TB GOLD in-tube assay (QFT) and the enzyme-linked immunosorbent assay (ELISA) where the release of IFN-γ, IFN-γ inducible protein 10 (IP-10), Interleukin 2 (IL-2) and Tumor Necrosis Factor-α (TNF-α) was determined in the whole blood with or without antigen-stimulation. The positive rates of the QFT, IP-10 and IL-2 tests were 86.4%, 89.4% and 86.4% for the ATB group with no difference between them (p>0.05). However, QFT in combination with IP-10 and IL-2 significantly increased the detection rate to 95.5% in the ATB group (p = 0.03) and the indeterminate rate of all samples decreased from 2.3% (5/215) to 0.4% (1/215). The un-stimulated level of IP-10 was significantly higher in the HHC than the ATB and HC groups. The IP-10 responses were strongly associated with extended *Mtb* exposure time and the degree of smear-positivity of the index cases. The IL-2/IFN-γ ratio in the antigen-stimulated plasma could discriminate LTBI from ATB with a sensitivity of 77.2% and a specificity of 87.2%.

**Conclusion:**

The increased *Mtb*-specific antigen-stimulated expression of IP-10 and IL-2 may be useful for detecting both ATB and LTBI. Combining the QFT with IP-10 and IL-2 could increase the detection accuracy of active TB over the QFT alone.

## Introduction

The *Mycobacterium tuberculosis* (*Mtb*)-specific T-cell interferon-gamma release assays (IGRAs) contribute greatly to the diagnosis of tuberculosis (TB), as they are capable of detecting *Mtb* infection with a higher sensitivity and specificity than the traditional tuberculin skin test (TST) [1], [2], [3], [4]. The IGRAs can discriminate *Mtb* infection from bacillus Calmette-Guérin (BCG) vaccination or exposure to non-tuberculous mycobacteria (NTM) by measuring IFN-γ response to *Mtb*-specific antigens (6-kDa early secretory antigenic target -ESAT-6, 10-kDa culture filtrate antigen-CFP-10 and Rv2654). However, the two current commercially available IGRAs, the QuantiFERON TB test (Cellestis, Victoria, Australia) and T-SPOT TB test (Oxford Immunotec, Abington, UK), fail to distinguish active disease from latent tuberculosis infection (LTBI), and especially have suboptimal detection rate and considerable indeterminate results in immunosupressed patients [3], [5], [6], [7]. Only measuring IFN-γ response with IGRAs may leave out other key steps of T-cell immune response to *Mtb* infection. Alternative or additional biomarkers are being investigated to enhance the diagnostic performance of the IGRAs for possible differentiation between LTBI and active TB (ATB).

IFN-γ-inducible protein of 10 kDa (IP-10, CXCL10), a member of the CXC chemokine family, is secreted by several cell types including monocytes, neutrophils, endothelial cells and fibroblasts. IP-10 expression can be highly induced mainly by IFN-γ but also by other cytokines like IL-2, IFN-α, IFN-β, IL-27, IL-17, IL-23, TNF-α and IL-1b.IP-10 is considered to function as a chemoattractor for monocytes and T cells at inflammatory foci [8], and it has been observed that its plasma level increased in patients with ATB and significantly reduced upon successful TB treatment, which could be useful in monitoring the disease activity and efficacy of therapy [9], [10]. Moreover, several studies have shown that antigen-stimulated IP-10 response as a potential diagnostic biomarker has a similar sensitivity as QFT in detecting ATB [11], [12], [13], [14], [15], [16]. However, few studies were conducted on the performance of IP-10 for detecting LTBI in subjects with high risk of TB exposure, especially in a TB-endemic and BCG-vaccinated area.

Interleukin-2 (IL-2) promotes T cell replication and is essential for cellular immunity and granuloma formation in *Mtb* infection. Several studies have demonstrated that IL-2 release stimulated by TB-specific antigens were significantly higher in TB patients than healthy controls and suggested that IL-2 could be a potential biomarker for diagnosing TB infection [17], [18], [19], and for discriminating ATB and LTBI when tested with a long incubation time (72 h) [20], though other study indicated that IL-2 might not be useful as a stand-alone diagnostic biomarker for TB infection due to its low amount in release [21]. In addition to ELISA or luminex®, other methods such as quantitative PCR [22], [23] and flow-cytometry [24] were also evaluated to detect antigen-specific IL-2 response. The potential of IL-2 alone or in combination with other biomarkers for diagnosing ATB and LTBI needs to be further evaluated.

Tumor Necrosis Factor-α (TNF-α) is another important cytokine which plays a central role in the control and protection against *Mtb* as IFN-γ. Recent studies revealed that the proportion of single-positive TNF-α *Mtb*-specific CD4+ T cells is a new tool for the rapid diagnosis of active tuberculosis disease [25]. The diagnostic value of TB antigen stimulated TNF-α in discriminating ATB from LTBI is still need to be further investigated.

We conducted a cross-sectional study to assess the diagnostic potential of IP-10, IL-2 and TNF-α for detecting ATB and LTBI in the TB-pandemic and BCG-covered regions. We also compared the results of IP-10, IL-2 and TNF-α with QFT and TST to determine whether the diagnostic performance of QFT could be improved by combining with the new biomarkers.

## Materials and Methods

### Study Population

This study received ethical approval from Institutional Review Board (IRB) of Huashan Hospital, Fudan University. Informed written consent was obtained from all the participants.

From January 2009 to September 2011, a total of two hundred and fifteen HIV-negative blood samples were included in this study from three groups of subjects: 66 active TB patients (ATB group), 73 household contacts (HHC group) and 76 healthy controls (HC group). All the blood donors aged 18 and older and had given their informed consent before blood collection.

The 66 patients with active pulmonary TB were recruited from 2 pulmonary disease hospitals in China. All of the subjects were diagnosed on extensive clinical evaluation of TB, including TB contact history, clinical manifestations, sputum smear of acid-fast bacilli (AFB), culture of *Mtb* and chest radiography. Among them, 47 (71.2%) were confirmed by microbiological examination based on the positive result of both culture of *M. tuberculosis* from sputum and smear microscopy for AFB and 19 (28.8%) patients with only positive smear microscopy for AFB. The patients were classified as having +, ++ and +++ AFB (http://www.who.int/tb/laboratory/en/) in sputum. To minimize the effect of anti-TB treatment on T-cell response, only patients on standard anti-TB therapy for <1 weeks were included in the study.

The HHC group consisted of 73 relatives of the patients in the ATB group. All the relatives were close TB contacts and lived in the same household with smear positive TB patient who was diagnosed no more than 3 months before recruitment of the contacts. They had no clinical symptoms or abnormal chest X-ray indicating ATB. According to their exposure time to the index case in hours per month, we classified the group into close contacts with a total exposure time of 90 hours or more (3 hours per day) and occasional contacts with exposure of less than 90 hours.

The 78 healthy adults in HC group were recruited from students and staff at Fudan University, China. All the subjects answered a detailed questionnaire concerning risk factors for exposure to *Mtb* and only those with no clinical and radiographic evidence of ATB and no known history of exposure to TB were enrolled.

### IP-10, IL-2 and TNF-α Determination

The levels of IP-10, IL-2 and TNF-α were measured in the plasma acquired from the QFT assay which was stimulated with saline, mitogen or TB-antigens (ESAT-6, CFP-10 and TB7.7). The Duoset ELISA Development kit (R&D Systems Inc, MN, USA) was used to detect IP-10, IL-2 and TNF-α release according to the manufacturer’s instructions. The plasma was diluted 1∶2 for IL-2 and TNF-α and diluted 1∶10 for IP-10 measurement. All the plasma specimens were tested in duplicate and expressed in pg/ml.

### QuantiFERON TB GOLD In-tube and IFN-γ Determination

The QFT test was performed according to the manufacturer’s instructions. Briefly, 1 ml of whole blood was drawn into three QFT tubes coated with saline (Nil Control), a peptide cocktail simulating ESAT-6, CFP-10 and TB7.7 proteins (TB Antigen) or PHA (Mitogen Control) respectively and incubated at 37°C as soon as the blood was collected (within 8 hours after collection). Following a 20 hour incubation period, the tubes are centrifuged and the plasma was harvested from each tube to determine the concentration of IFN-γ. The remaining plasma was stored at −80°C for testing IP-10 or IL-2. QFT tests were regarded as positive if the antigen-stimulated response of IFN-γ (TBAg-Nil) was ≥0.35I U/ml (17.5 pg/ml), or negative if the mitogen-stimulated response (Mitogen-Nil) was ≥0.5 IU/ml (25 pg/ml) and the antigen-stimulated response was <0.35I U/ml, or indeterminate if both mitogen-stimulated and antigen-stimulated responses were <0.35 IU/ml or un-stimulated response (Nil) >8 IU/ml (400 pg/ml). The level of IFN-γ was presented as pg/ml to facilitate comparisons with the other biomarkers detected. One International unit of IFN-γ corresponds to 50 pg/ml (NIBSC, Potters Bar, UK).

### Tuberculin Skin Test (TST)

TST was performed on the household contacts (HHC group) at the time of initial assessment. Five tuberculin units (TU) of purified protein derivative (PPD) RT23 (Statens Serum Institute, Copenhagen, Denmark) was applied intradermally in the medium third of the anterior surface of the left forearm by Mantoux method. After 72 hours, the largest transverse diameters of the palpable hardened areas were measured with a millimeter ruler. The results were read by two trained professionals. For this study, it was classified as negative if TST induration was smaller than 5 mm in diameter, or positive if equal to or greater than 5 mm in diameter or there was blistering.

### Statistical Analysis

The differences in levels of biomarkers among groups were analyzed using non-parametric analysis of variance with the Kruskall-Wallis test. The difference between paired proportions was tested by McNemar’s test. The degree of correlation between the biomarkers was assessed with the Spearman rank test. Test concordance was assessed using the kappa (κ) statistic [26]. Multivariate analysis was performed to identify factors associated with positive results of each test. A *P*-value of <0.05 was considered significant. Diagnostic accuracies of the tests were evaluated using receiving operating characteristic (ROC) curve. The cut-off values were estimated at various sensitivities and specificities and determined at the maximum Youden’s index (YI), *i.e.* sensitivity + specificity –1 [27].The statistical analysis was performed using the GraphPad Prism V5.03 software (GraphPad, San Diego, USA).

## Results

### Characteristics of the Enrolled Individuals

A total of 215 subjects were included in the final analysis, which consisted of 66 patients with ATB, 73 household contacts and 76 healthy controls. The demographic and clinical characteristics of participants in this study are shown in Table 1. There were no significant differences among the groups regarding age or gender. BCG scar was found in 52 (78.9%) TB patients, 54 (74.0%) HHC and 68 (89.5%) HC.

**Table 1:** Demographic and clinical characteristics of the study population.

	ATB	HHC	HC
Total no.	66	73	76
Male, n (%)	39(59.1)	35(47.9)	40(54.8)
Median age (range), years	45(16–86)	41(18–83)	38(18–50)
BCG vaccinated, n (%)	52(78.9)	54(74.0)	68(89.5)
Presence of TB history, n (%)	6(9.0)	0(0)	0(0)
Cavity on chest x-ray, n (%)	30(57.7)	0(0)	0(0)
Smear grade			
+	42(57.5)	–	–
++	11(15.1)	–	–
+++	20(27.4)	–	–
TST results, n (%)			
Induration <5 mm	–	42(57.5)	–
Induration, 5–10 mm	–	11(15.1)	–
Induration, >10 mm	–	20(27.4)	–
Exposure time ≥90 hours	–	57(78.1)	–

ATB, subjects with active tuberculosis; HHC, household contact; HC health control.

In the QFT, 57 of 66 patients with ATB were QFT-positive (86.4%). In HHC and HC group, the positive rate was 38.4% (28/73) and 17.1% (13/76) respectively. However, 3 (4.5%) in ATB group, 1(1.4%) in HHC group and 1(1.3%) in HC group had indeterminate QFT results, all due to low mitogen response except for one in HHC group whose IFN-γ level was >8.0 IU/ml (400 pg/ml) in un-stimulated control.

TST results were available only in HHC group and the positive rate was 42.5% (31/73), which was higher than that of QFT test (38.4%, 28/73) though the difference was not significant (p = 0.557). The agreement between TST and QFT test in HHC group was relatively low (46/73, 63.0%, κ: 0.233).

### IFN-γ, IL-2 and IP-10 Levels Measured by ELISA

There was a high correlation in TB antigen-stimulated response between IP-10 and IFN-γ (r = 0.7801, p<0.0001) and between IL-2 and IFN-γ (r = 0.8032, p<0.0001). However, in mitogen-stimulated samples, the correlation was not so obvious for both IP-10 (r = 0.1609, p = 0.0182) and IL-2 (r = 0.4145, p<0.0001), though it was still statistically significant. There was no correlation observed between TNF-α and the other biomarkers in both TB antigen and mitogen stimulated response.

**Figure 1:**
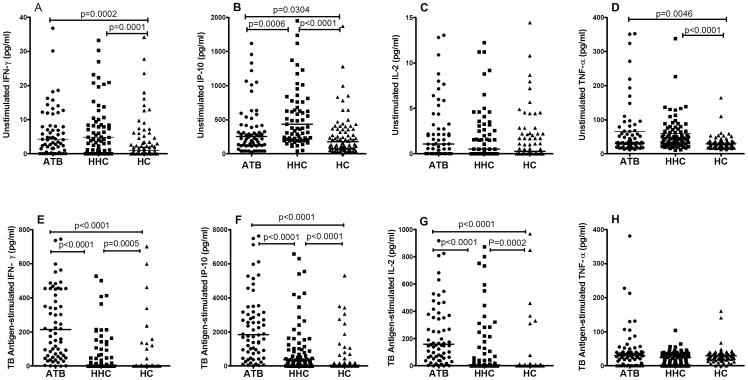
The un-stimulated and TB antigen-stimulated expression of IFN-γ, IP-10, IL-2 and TNF-α in patients with active tuberculosis (ATB group), household contacts (HHC group) and healthy controls (HC group). The expression of IFN-γ (A and E), IP-10 (B and F), IL-2 (C and G) and TNF-α (D and H) were determined using ELISA and expressed in pg/ml. The horizontal line indicates the median amount of biomarker production.

The antigen-stimulated expression of IFN-γ, IP-10 and IL-2 from high to low in the three groups were: ATB, HHC and HC, and the difference between any two groups were significant (Figure 1E–G, Table 2). The un-stimulated IFN-γ release was significantly lower in the HC group than the ATB and HHC group (Figure 1A, Table 2). Interestingly, the un-stimulated release of IP-10 from high to low followed: HHC, ATB, and HC, and the difference between each two groups were significant (Figure 1B, Table 2). In the mitogen-stimulated level, we observed that mitogen stimulated significantly higher levels of all the three biomarkers in HC group than ATB and HHC group. TB antigen-stimulated TNF-α release was generally low and no significant differences were found among the three groups (Figure 1H, Table 2). The un-stimulated TNF-α release was significantly lower in the HC group than the ATB and HHC group (Figure 1D, Table 2).

**Table 2:** Levels of IFN-γ, IP-10, IL-2 and TNF-α released in un-stimulated, TB antigen-stimulated and mitogen-stimulated plasma.

Cytokine(pg/ml)	ATB (n = 66)	Stratified by risk of exposure		Stratified by QFT results	
		HHC (n = 73)	HC (n = 76)	LTBI (n = 41)	NTB (n = 108)
**IFN-γ**					
Un-stimulated	4.3(1.3–11.9)	4.8(0.7–11.6)	0.9(0.0–4.3)a,d	4.6(1.1–8.9)	1.3(0–6.1)^b,e^
TB antigen-stimulated	213.5(59.3–454)	4.1(0.0–79.7)^a^	0.0(0.0–2.2)a,d	158.3(10.6–386.1)	0(0–1.5)a,d
Mitogen-stimulated	598.0(293.3–1191)	514.4(426.3–1012)	1128(962.6–1414)a,d	626.2(435.6–1241)	1026(651.7–1240)a,f
**IP-10**					
Un-stimulated	252.4(123.9–448)	433.4(216.3–847.6)^a^	175.2(77.4–360.4)^c,d^	446.8(207.1–1559)^b^	253.1(107.5–484.8)^e^
TB antigen-stimulated	1846.0(769.2–3163)	388.2(108.2–1453)^a^	50.4(0.3–188.6)a,d	1876(788.4–3478)	66.5(0–24.6)a,d
Mitogen-stimulated	2591(1150–4902)	4571(2776–6754)^a^	3451(1700–12056)^c^	4361(2548–7127)^b^	4041(1801–6974)^b^
**IL-2**					
Un-stimulated	1.1(0.0–4.4)	0.5(0.0–3.1)	0.3(0.0–2.2)	0(0–1.9)	0.5(0–3.2)
TB antigen-stimulated	159.7(62.2–393.7)	7.0(1.0–132.7)^a^	1.1(0.0–6.3)a,d	283.2(63.6–572.5)	1.1(0–4.7)a,d
Mitogen-stimulated	400.7(147.2–757.2)	862.4(364.0–1185)^a^	2372(2015–3228)a,d	1082(659.4–1603)^a^	1447(764.0–2162)^a^
**TNF-α**					
Un-stimulated	32.2(21.5–66.7)	44.3(28.0–74.6)	27.1(19.5–36.6)a,d	42.5(19.3–68.2)	30.5(23.2–42.3)^b,e^
TB antigen-stimulated	30.4(11.7–40.9)	24.9(4.2–35.5)	29.1(20.7–35.4)	28.4(6.4–37)	28.3(13.4–35.6)
Mitogen-stimulated	601.1(275.3–1383)	1242(872.3–1655)	2288(1682–2714)a,d	1354(679.4–1830)	1865(894–2172)a,f

Data are presented as median concentration in pg/ml (interquartile range).

Statistical significance was determined versus ATB group,

a p<0.001;^b^0.001≤p<0.01;^c^0.01≤p<0.05.(Kruskal-Wallist test).

Statistical significance was determined between HC and HHC group or between NTB and LTBI group, ^d^: p<0.001; ^e^: 0.001≤p<0.01; ^f^: 0.01≤p<0.05. (Kruskal-Wallist test).

We also compared the levels of the three new biomarkers in groups stratified by QFT results. The QFT-positives in the HHC and HC groups were regrouped as latent tuberculosis infection (LTBI) group and the QFT-negatives in these two groups was regarded as no TB infection (NTB) group. As shown in Table 2, significantly higher levels of TB antigen-stimulated IFN-γ, IP-10 and IL-2 were observed in ATB group and LTBI group than NTB group. However, no significantly difference was observed in antigen-stimulated levels of three cytokines between ATB and LTBI group.

The un-stimulated levels of IP-10 from high to low in these three groups were: LTBI, ATB, and NTB, in which there was a significant difference between the LTBI and the other two groups, but no significant difference between the other two groups (p = 0.4756) (Table 2). When we stratified the LTBI group into subjects from HHC and HC group, we observed a significantly higher level of baseline IP-10 in the HHC with LTBI (median: 395.2 pg/ml, interquartile range: 107.6–1440 pg/ml) than in the HC with LTBI (median: 153.1 pg/ml; interquartile range: 60.5–573.9 pg/ml p = 0.0179).

### Diagnostic Performance of IP-10 and IL-2

The diagnostic potential of the three biomarkers in the TB-antigen stimulated experiment was first assessed with the ROC curve. The ATB patients were used as the true-positives and QFT-negative healthy controls as true-negatives. The AUC of IFN-γ, IP-10 and IL-2 were 0.9749 (95%CI: 0.9461–1.004), 0.9594 (95%CI: 0.9278–0.9909) and 0.9555 (95%CI: 0.9147–0.9963), respectively. There was no significant difference of AUC between each pair of them.

**Figure 2:**
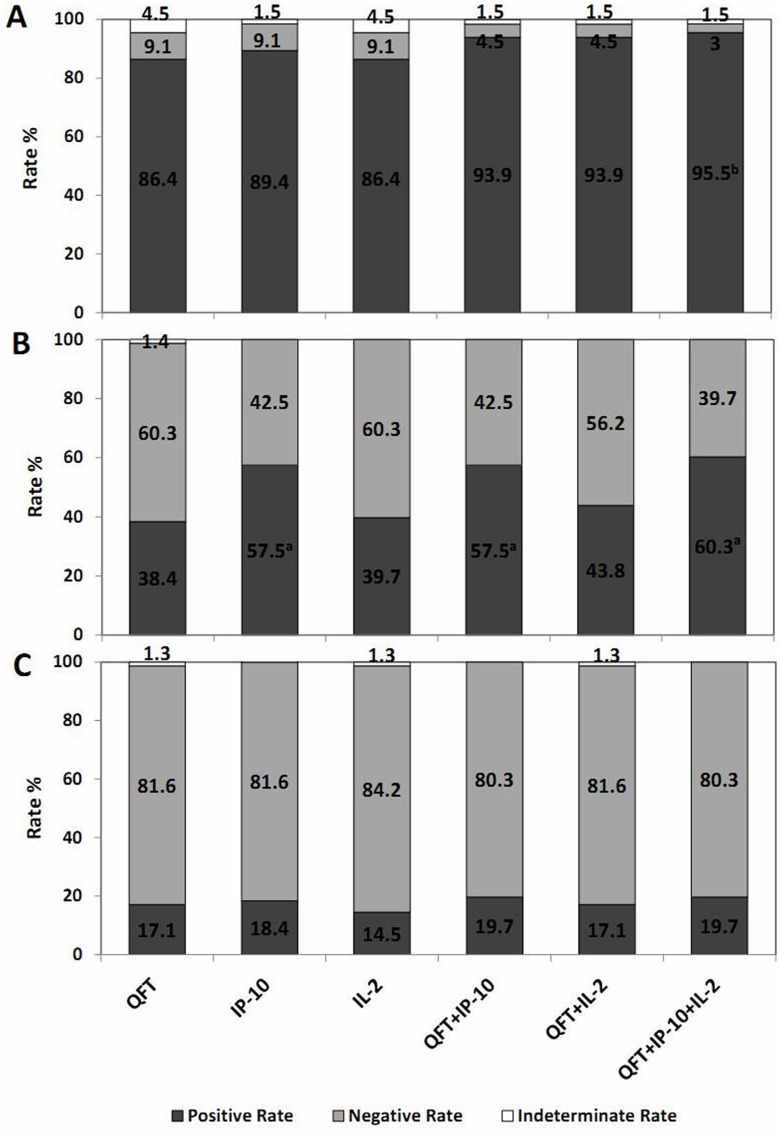
Distribution of positive, negative and indeterminate responders with the QFT, IP-10 and IL-2 test and when the tests are combined: (A) active tuberculosis (ATB group), (B) household contacts (HHC group) and (C) healthy controls (HC group). In the combination test, a positive responder was determined if any of the tests was positive. All the detection rate were compared with QFT by McNemars test, a: p<0.001, b: p<0.05.

The optimal cut-off values for IP-10 and IL-2 test were also determined by the ROC analysis, to be 451.3 pg/ml for IP-10 and 13.1 pg/ml for IL-2. Those with antigen-stimulated level ≥ the optimal cut-off were regarded as positives, and those below the cut-off were otherwise regarded as negatives, and indeterminate responder was determined if the antigen-stimulated response was negative and the mitogen-stimulated response was <200 pg/ml for the IP-10 test [12] and <50 pg/ml for the IL-2 test. These cut-offs were arbitrarily chosen. The results of the three tests were shown in Figure 2. In ATB group, the positive rate of the IFN-γ, IP-10 and IL-2 was 86.4% (95%CI: 75.9–92.9), 89.4% (95% CI: 79.4–95.1) and 86.4% (95% CI: 75.9–92.9), respectively, with no difference between any two groups. The indeterminate rate was 4.5% (3/66) in the QFT, 1.5% (1/66) for IP-10 test and 4.5% (3/66) for IL-2 test. Notably, in the HHC group, the positive rate of IP-10 was higher than the QFT (p<0.0001) and IL-2 test (p<0.0001), but no significant difference between the QFT and IL-2. The QFT, IP-10 and IL2 test in the HC showed the similar positive rates, 17.1%, 18.4% and 14.5%, respectively. Of all the 215 subjects tested, the indeterminate rate of QFT, IP-10 and IL-2 test was 2.3% (5/215), 0.4% (1/215) and 1.9% (4/215), respectively.

The head-to-head comparison and concordance between the different tests in ATB, HHC and HC group are shown in Table 3. In total, there was a high agreement between the IP-10 and QFT test (186/215, 86.5%, κ 0.739) and the IL-2 and QFT test (195/215, 90.7%, κ 0.820). The three tests agreed in 179 out of 215 subjects (83.3%) in total. The agreement between TST and IP-10 or IL-2 in the HHC group was also low, which was 52.1% (38/73, κ 0.062) and 64.4% (47/73, κ 0.265), respectively.

**Table 3:** Comparison of responder numbers and agreement among the QFT, IP-10 and IL-2 tests in the ATB, HHC and HC group.

QFT test	IP-10 test					IL-2 test				
	Positive	Negative	Indeterminate	Total	Kappa,Agreement (%)	Positive	Negative	Indeterminate	Total	Kappa,Agreement (%)
**ATB group**										
Positive	54	3	0	57	0.377, 86.4	52	3	2	57	0.379, 84.8
Negative	4	2	0	6		3	3	0	6	
Indeterminate	1	1	1	3		2	0	1	2	
Total	59	6	1	66		57	6	3	66	
**HHC group**										
Positive	27	0	0	27	0.600, 78.1	24	3	0	27	0.794, 89.0
Negative	15	30	0	45		4	41	0	45	
Indeterminate	0	1	0	1		1	0	0	1	
Total	42	31	0	73		29	44	0	73	
**HC group**										
Positive	12	1	0	13	0.865, 94.7	11	2	0	13	0.909, 97.4
Negative	2	60	0	62		0	62	0	62	
Indeterminate	0	1	0	1		0	0	1	1	
Total	14	62	0	76		11	64	1	76	

### Risk Factors Associated with Positive Results

We performed logistic regression analysis to evaluate the association between the positive results and potential risk factors for *Mtb* infection in the HHC (Table 4). The independent variable “exposure≧90 hours” was associated with the positive result of QFT, IP-10 and IL-2 test with highly statistically significant odds ratios (p = 0.031, 0.003 and 0.047 respectively), while there was no association obtained by TST test. In addition, the IP-10 test was significantly increased among subjects exposed to active TB patients with high smear positive grade. Similar trend was found within QFT and IL-2 test though it was not as obvious as that in IP-10 test. Besides, positive IL-2 test was associated with gender and age (Table 4).

**Table 4:** Logistic regression analysis of the association of positive results of QFT, IP-10, IL-2 and TST with potential risk factors relevant to *Mtb* infection in HHC group.

Risk factor	QFT		IP-10		IL-2		TST	
	Adjusted OR (95%CI)	P-value	Adjusted OR (95%CI)	P-value	Adjusted OR(95%CI)	P-value	Adjusted OR (95%CI)	P-value
Sex, Male	1.2(0.4–3.3)	0.765	0.9(0.3–2.5)	0.794	3.2(1.0–10.0)	***0.048***	2.3(0.9–6.2)	0.1
Age ≥ median age^a^	1.0(0.9–1.05)	0.212	1.0(0.9–1.03)	0.842	1.04(1.0–1.07)	***0.035***	1.0(0.9–1.03)	0.999
BCG vaccination	0.6(0.2–2.2)	0.471	1.3(0.4–4.4)	0.684	0.4(0.1–1.7)	0.236	1.1(0.3–3.6)	0.886
Exposure ≥90 hours	4.5(1.0–22.5)	***0.031***	7.9(2.0–32.0)	***0.003***	4.8(1.0–24.1)	***0.047***	0.8(0.3–2.7)	0.824
Smear grade								
++	4.1(0.8–21.8)	0.094	7.6(1.7–32.8)	***0.007***	3.2(0.6–17.6)	0.176	0.8(0.2–2.9)	0.811
+++	5.3(1.0–29.6)	0.058	8.5(1.8–40.5)	***0.007***	8.9(1.5–53.3)	***0.02***	0.8(0.2–3.3)	0.843

OR: odds ratio; 95%CI: 95% confidence interval.

a Median age: 41 years old, the median age calculated in HHC group.

### Improved Performance by Combining the Biomarkers

We further assessed the diagnostic performance when the QFT was combined with the IP-10 or/and IL-2. In the combination experiments (Figure 2), a positive responder was determined if any of the tests was positive. In the ATB group, the detection rate was increased to 93.9% (95%CI: 85.0–98.1) when the QFT was combined with IP-10/IL-2 test, and even up to 95.5% (95%CI: 87.0–99.0) when combined with both biomarkers. There was a statistically significant difference in the detection rate between the three tests combined and QFT alone (p = 0.031).

In the HHC group, the positive rate significantly increased to 57.5% (42/73) (p<0.0001) and 60.3% (44/73) (p<0.001) in the combination of QFT+IP-10 and QFT+IP-10+IL-2, respectively, compared with QFT at 38.4% (28/73). Meanwhile, the positive rate of any combination in the HC group remained low and similar to that of the QFT alone (Figure 2).

The indeterminate rates of the QFT in the three groups were all reduced upon the combination (Figure 2). Totally, QFT+IP-10 had the lowest indeterminate rate (0.5%, 1/215) but without significant difference when compared to the QFT (2.3%, 5/215). The addition of IL-2 did not help to further reduce the indeterminate rate for QFT+IP-10 test.

### Discrimination of LTBI from ATB by IL-2/IFN-γ Ratio

**Figure 3:**
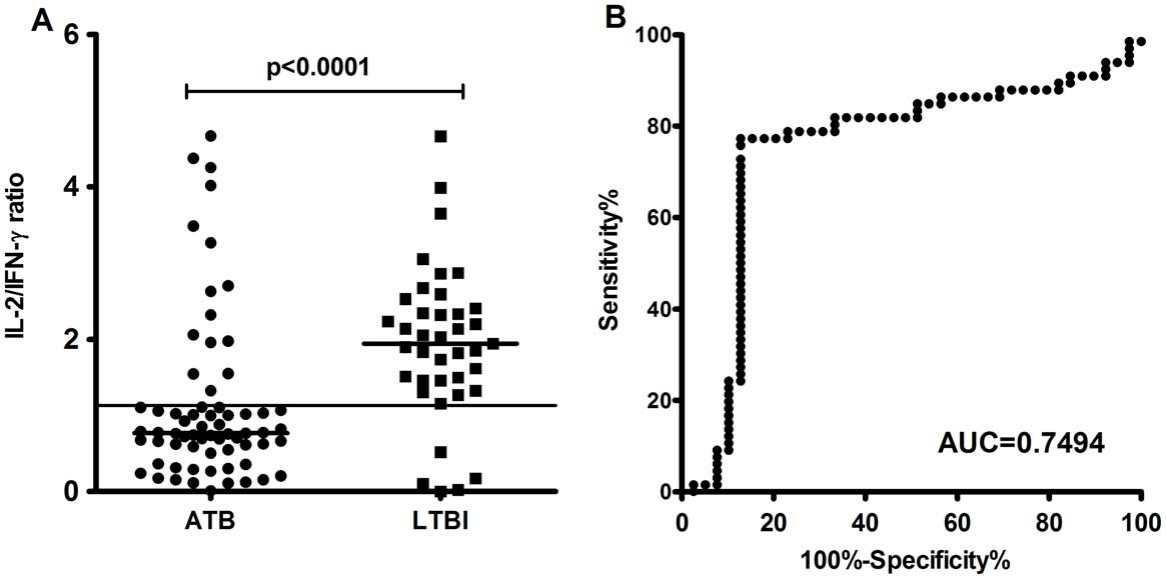
IL-2/IFN-γ ratio in subjects with active TB (ATB group) and in subjects with latent TB infection (LTBI group). A. IL-2/IFN-γ ratio in response to TB antigens was significantly higher in LTBI group than in the ATB group (p<0.0001). Horizontal lines indicate the median IL-2/IFN-γ ratio. B. The ROC analysis using subjects with active TB as patients and subjects with latent TB as controls.

The ratio of different cytokines was subjected to analysis and showed that the IL-2/IFN-γ ratio was closely related to the latent or active status of the disease (Figure 3). The antigen-stimulated IL-2/IFN-γ ratios in LTBI subjects were significantly higher than those in active TB patients (p<0.0001). The AUC was 0.7494 (95% CI: 0.6419–0.8569, P<0.0001) by the ROC analysis and the threshold of 1.133 gave a sensitivity of 77.2% and a specificity of 87.2% for ATB. The logistic regression analysis also shown that the positive IL-2/IFN-γ ratios are associated with risk factors for active TB in HHC group including the variable “exposure≧90 hours” (OR:6.7, 95%CI:1.0–23.9; p = 0.029) and “smear grade +++” (OR:4.8, 95%CI:1.0–12.5; p = 0.037). The ratios of the other biomarkers were also analyzed, but no significant difference was found between ATB and LTBI group in either antigen-stimulated or mitogen-stimulated release.

## Discussion

### Performance Characteristics of the New Biomarkers

This study evaluated the diagnostic performance of IP-10 and IL-2 in diagnosing *Mtb* infection in TB endemic and BCG-vaccinated regions. Our results demonstrated that the levels of IP-10 and IL-2 were significantly increased following stimulation of *Mtb*-specific antigens in both ATB and LTBI subjects (Figure 1/Table 2), which indicated two promising biomarkers to detect TB infection and disease. In combination with IP-10 and IL-2 test, the diagnostic performance of QFT in detecting *Mtb* infection was enhanced with a higher sensitivity and lower indeterminate rate. However, there was no significant difference in antigen-stimulated changes of both biomarkers between ATB and LTBI group (Table 2). There was inconsistency in antigen-stimulated changes of IP-10/IL-2 from different studies [21], [28], due probably to differences in patient characteristics, detection methods and antigens used for stimulation. The QFT, IP-10 and IL-2 tests rendered similar positive rates in the HC group with low risk of *Mtb* exposure (17.1%, 18.4% and 14.5%, respectively), which suggested that those antigen-stimulated positives in health controls could result from LTBI rather than false positive responders, as the investigated population from an area with high prevalence of TB [29], [30].

The IP-10 test alone or combined with the QFT seemed to identify more positive subjects in the HHC group than the QFT alone did. Using a 450 pg/ml cut-off point, the detection rate of IP-10 test in HHC group was higher than that of QFT or IL-2 test (Figure 2). Similar results were observed in other studies [12], [22], [31]. Since there is no gold standard for detecting LTBI and the sensitivity of IP-10 test for diagnosing LTBI cannot be evaluated directly, we evaluated the association of the risk factors of TB infection with IP-10-positives. It turned out that IP-10-positivity was significantly associated with risk factors of TB infection such as long exposure time and the degree of smear-positivity of the index cases (Table 4). On the other hand, it is not sufficiently explored if positive IP-10 test in household contacts will be sensitive predictors of progression to active TB disease in this study.

Tuberculin skin test (TST) has been widely used for diagnosis of latent tuberculosis infection (LTBI). Purified protein derivative (PPD), the antigens used by TST, is a crude mixture of *M. tuberculosis* antigens and some of these antigens are cross-reactive with BCG and environmental mycobacteria [2]. The observations of a low agreement between QFT and TST in the HHC (63.0%;κ = 0.233) and no correlation between the TST-positive results with the risk factors for TB infection (Table 4) indicated that the performance of TST may be attenuated by the high coverage of BCG vaccination and high prevalence of NTM [29], [30], and accordingly LTBI incidence in China may be inappropriately estimated just relying on the TST. Since the release of IP-10 and IL-2 both correlates with IFN-γ, it is not surprising that a low agreement was also observed between the TST and IP-10 (52.1%; κ = 0.062) or IL-2 test (64.4%; κ = 0.265).

### Baseline IP-10 Levels Associated with High Risk of Active TB

Parallel with several other studies [9], [10], [15], [22], [32], the elevated levels of baseline IP-10 were observed in ATB patients and household contacts but not in healthy controls indicating that IP-10 levels could be elevated with ongoing and enhanced activation of cells involved in the inflammatory response to *Mtb* infection [9]. Consistent with a recent study [13], our data also revealed a significantly higher level of baseline IP-10 in LTBI group than ATB group (Figure 2B), which may result from both the ongoing control of the subclinical infection with *Mtb* in LTBI and the immunosuppressive effect of cellular immunity in ATB. It is also noteworthy that there was a significantly higher level of baseline IP-10 in the HHC with LTBI than in the HC with LTBI. Without known exposure to recent TB infection, QFT-positives in the HC group were most likely a result of remote infection, while those in the HHC group probably had recent infection due to close contact with smear-positive pulmonary TB patients, and those QFT-positives with elevated level of baseline IP-10 may suggest a higher risk to develop ATB, for the recent finding showed that IP-10 level increased when exposed household contacts developed to active TB [9]. Since the current IGRAs are not able to identify those with recently acquired infection who have the highest risk of active tuberculosis, IP-10 response may serve to characterize recently infected cases with the highest risk of progression to active disease, which may receive the greatest benefit from preventive therapy. The diagnostic value of baseline IP-10 level in differentiating recent from remote infection should be further studied in a large scale of investigation.

### The IL-2/IFN-γ Ratio Reflects the Different Cytokine Profiles of TB-specific T cells in ATB and LTBI

Th1-type CD4+ T cells and relevant cytokines especially IFN-γ, IL-2 and TNF-α have been proposed as key components in the control of and protection against *Mtb* infection [33], [34]. A series of studies have recently revealed that dominant IL-2 functional T cell signatures were associated with antigen clearance. There were higher proportions of IL-2/IFN-γ double CD4+ T cells in LTBI or successfully treated TB subjects which have lower bacterial loads while in active TB the cytokine profile was shifted towards cells secreting IFN-γ only [35], [36], [37], [38], [39]. This is confirmed by our finding in the cytokine release assay that the antigen-stimulated IL-2/IFN-γ ratio was significantly higher in the LTBI group than in the ATB group with a dominant IFN-γ response (Figure 3). The optimum sensitivity of 77.2% and specificity of 87.2% of the IL-2/IFN-γ ratio in detecting ATB from LTBI (cut-off value of 1.133) suggested that this ratio may not function as a stand-alone diagnostic indicator of LTBI, but one of the valid methods to distinguish active TB from latent infection. On the other hand, compared with the common methods used to analyze the cytokine profile of *Mtb*-specific T cell subsets such as intracellular cytokine staining and flow-cytometry, direct detection of the ratio between cytokine releases may be more convenient and simpler to perform. A large-scale of study and prolonged incubation time is required to ascertain the diagnostic potential of the IL-2/IFN-γ ratio. A recent study investigating the different cytokines profiles including IFN-γ, IL-2 and TNF-α in *Mtb*-specific CD4+ T cells have shown that the proportion of single-positive TNF-α *Mtb*-specific CD4+ T cells was the strongest predictive measure of discrimination between active disease and latent infection [25]. Results from another study also shown TNF-α production following TB10.4 stimulation was higher from ATB than LTBI. These indicated a dominant TNF-α response in active TB patients compared with IFN-γ and IL-2. However, Consistent with some other studies [18], [19], [31], the TNF-α response stimulated by QFT antigens and an incubation time of 20 h was very low in our study and no significant difference of TNF-α/IFN-γ or TNF-α/IL-2 ratio were observed. Despite the different findings of TNF-α behavior in cell activation relevant to TB infection, the role of TNF-α in TB-specific response needs further clarification maybe using stronger antigens to stimulate in a longer incubation time [40], [41].

### The Limitations and Perspectives of the Study

The present study had some limitations. First of all, since there is no gold standard for LTBI, the QFT was used in our study as a diagnostic surrogate. It was not appropriate to use QFT as a diagnostic standard if it was also used as comparator with the other biomarkers. To reduce this bias, we did not compared the sensitivity of the biomarkers in LTBI group directly and chose to evaluate the diagnostic value of QFT, IP-10 and IL-2 for LTBI by calculating the association between the positive results and potential risk factors for *Mtb* infection instead. However, for the ROC analysis and cut-off determination, we were forced to include only QFT negative controls to maximally exclude subjects with LTBI, since LTBI can be detected even in a population with low risk of *Mtb* exposure in a high TB endemic area [29], [30]. This may bring a bias to the cut-off value and became the main reason why the cut-off in our study was different from another study (698 pg/ml) using the same detection method and dilution factor [42]. Longitudinal cohort studies will be required with careful clinical characterization of participants into TB infection and disease groups to validate the accuracies and the cut-off values of the markers identified in this study. Moreover, the cross-sectional design of our study could lead to the risk of over-optimistic cut-offs and overestimated test accuracy. Furthermore, the mitogen cut-off in our study was arbitrary chosen. An adjustment to the mitogen cut-off defining indeterminate results should ideally be made on evaluation for a possible immunocompromised state in low mitogen responder and on the assessment of technical conditions. Besides, it may be inappropriate to estimate the specificity of the biomarkers in our study population from a TB-endemic area. The test accuracy of the new biomarkers needs to be tested and verified in other populations with different TB endemic rates.

The future study should enlarge the sample size, recruiting more subjects especially those with LTBI and with immunodeficient conditions such as HIV infection and immunosuppressive therapy for rheumatoid arthritis. In fact, TB screening in these subjects is somewhat limited by the low detection rate and considerable indeterminate results of the current IGRAs [5], [6], [7]. Future prospective studies should be performed to validate the diagnostic value of the IP-10 and IL-2 in patients suspected of tuberculosis infection. A longitudinal study needs to be conducted in the future to determine the predictive value for progression to active tuberculosis of the two new biomarkers in a large cohort of subjects with high risk of TB infection. Additionally, simpler and less costly procedures are needed for measuring the new biomarkers in favor of application in those places with poor medical infrastructure. There are a couple of promising methods such as the detection of IP-10 in dried blood spots on filter paper [43], quantitative PCR detection of IP-10 mRNA based on a finger-prick volume of blood [44], [45]. Besides, simple field-friendly lateral flow methods for detecting the new biomarkers are also needed for large scale screening.

### Conclusion

The present study was based on a cross-sectional study conducted in active TB patients, subjects of latent infection and community healthy controls in a TB endemic, BCG-vaccinated area. We proved that *Mtb* specific antigen-stimulated increase in both IP-10 and IL-2 may be useful for detecting ATB and LTBI. Combining IP-10 and IL-2 with QFT may increase the detection accuracy and reduce the indeterminate results. The IL-2/IFN-γ ratio in the antigen-stimulated plasma has the potential to discriminate LTBI from ATB. We believed that these findings support the use of IP-10 or IL-2 as new biomarkers to facilitate the diagnosis of TB and LTBI.
